# Supplementary figures and images for: Preliminary Analysis of the Salt-Tolerance Mechanisms of Different Varieties of Dandelion (Taraxacum mongolicum Hand.-Mazz.) Under Salt Stress
Source: Curr Issues Mol Biol. 2025 Jun 11;47(6):449. doi: 10.3390/cimb47060449 (PMC12191469; doi:10.3390/cimb47060449)

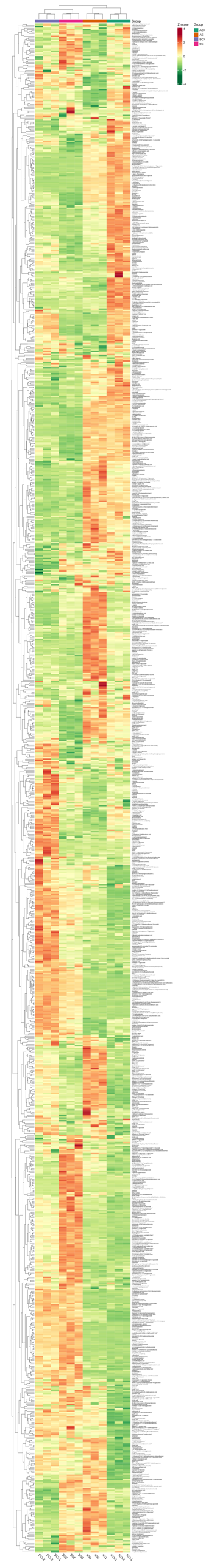

Supplement: Supplementary file 1 [file cimb-47-00449-s001.zip › Figure S1.png]

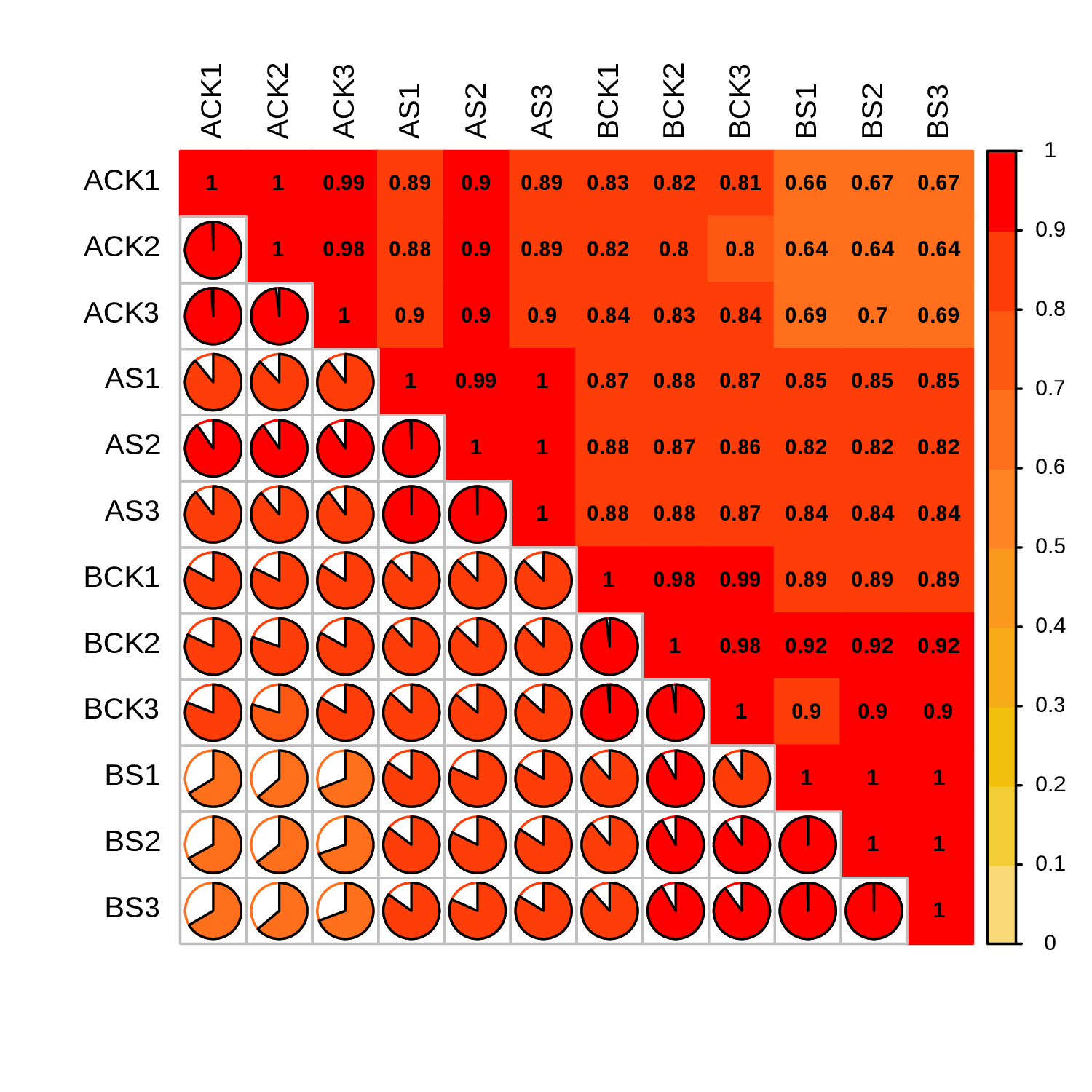

Supplement: Supplementary file 1 [file cimb-47-00449-s001.zip › Figure S2A.png]

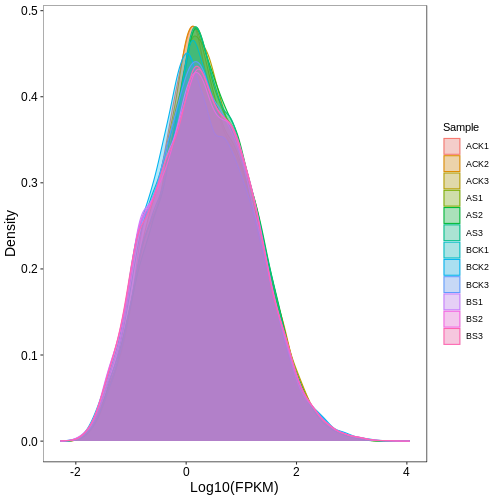

Supplement: Supplementary file 1 [file cimb-47-00449-s001.zip › Figure S2B.png]

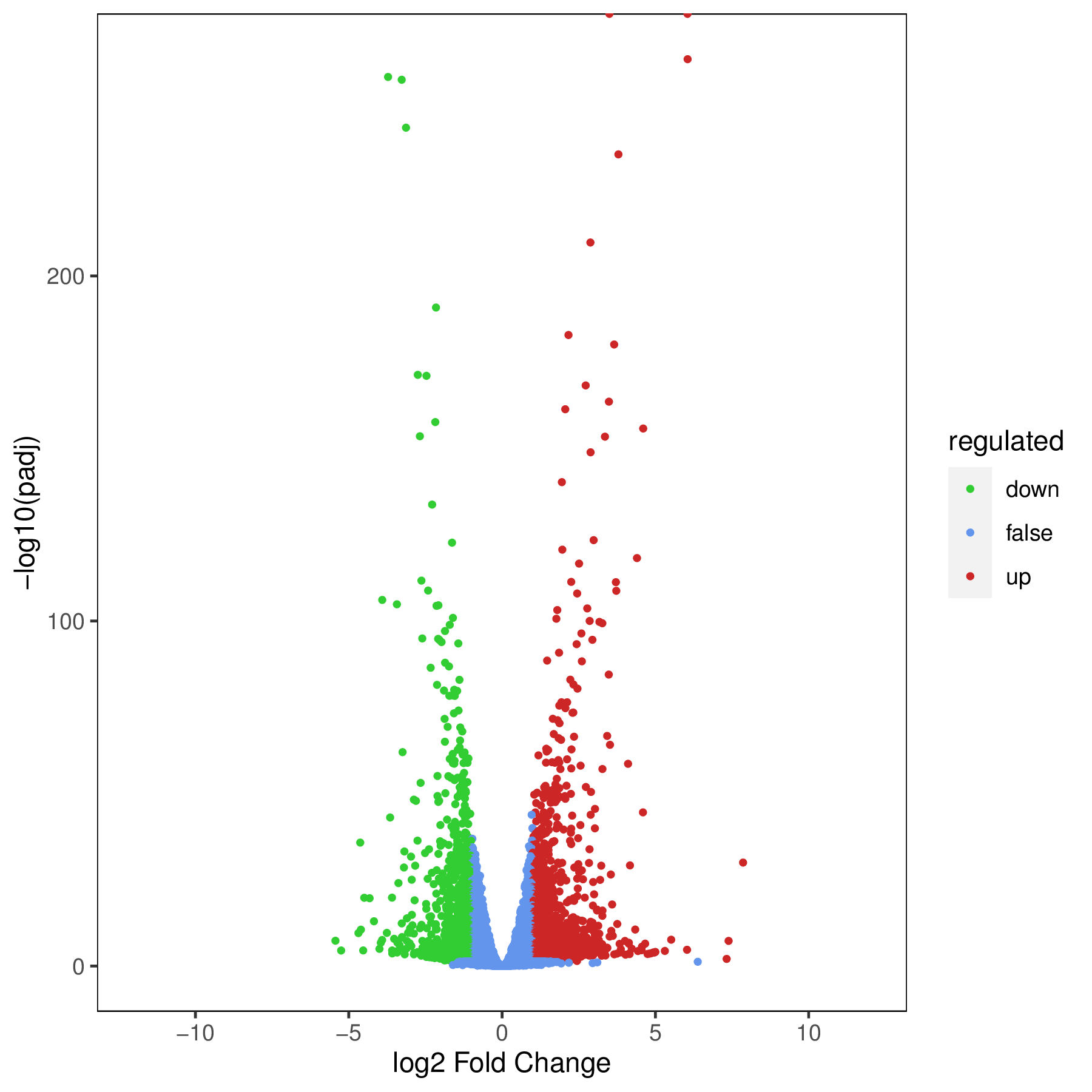

Supplement: Supplementary file 1 [file cimb-47-00449-s001.zip › Figure S3A.png]

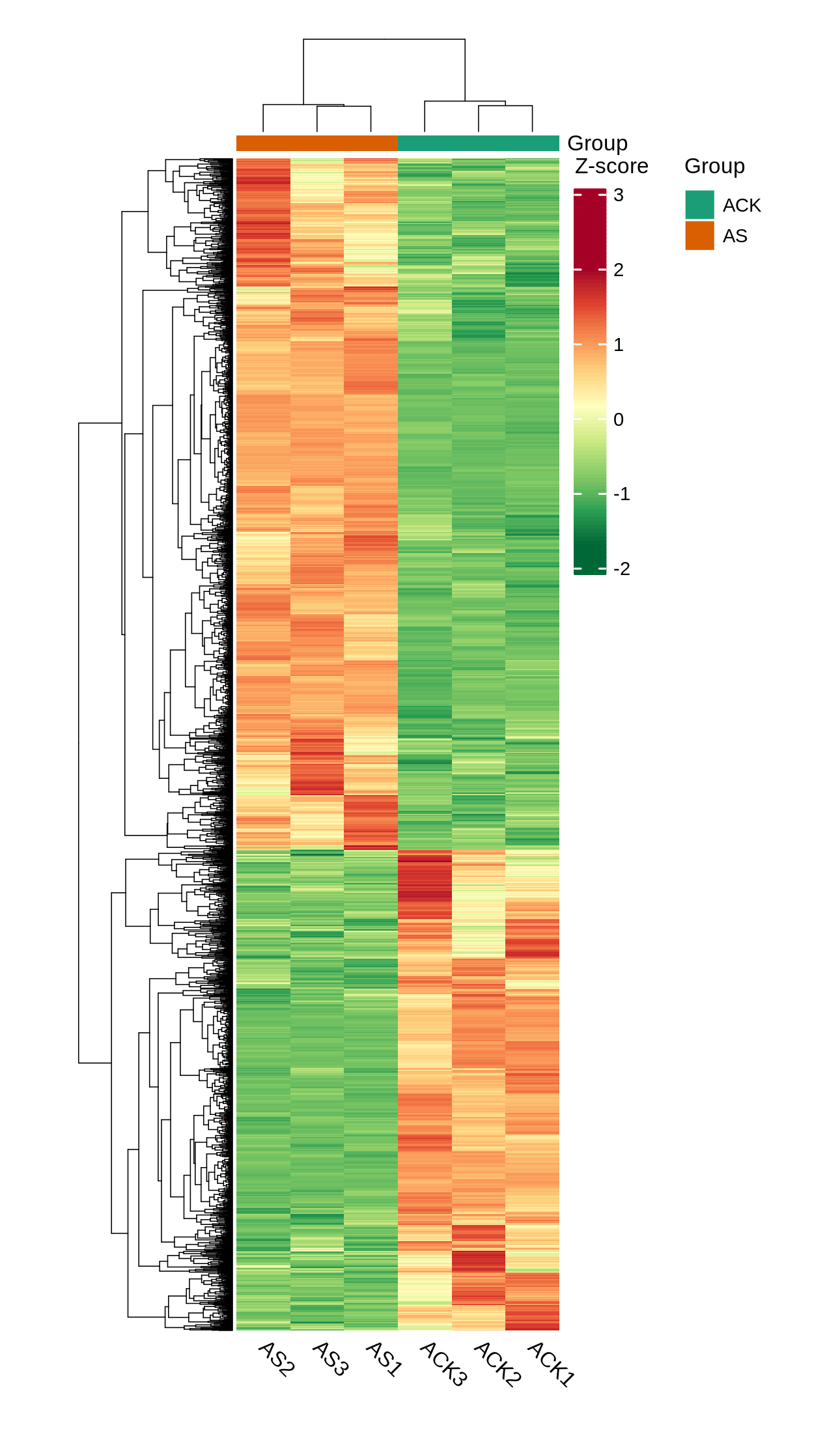

Supplement: Supplementary file 1 [file cimb-47-00449-s001.zip › Figure S3B.png]

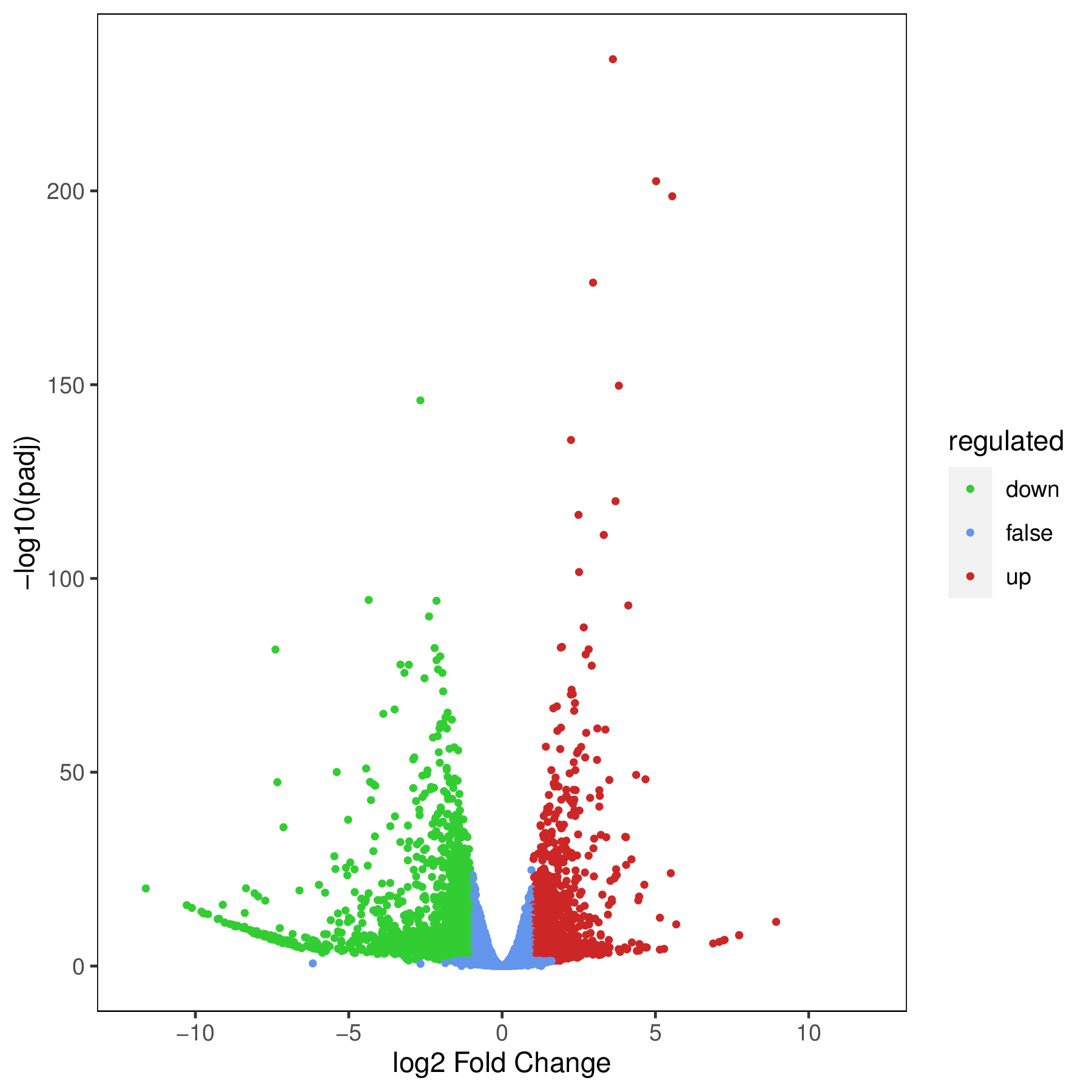

Supplement: Supplementary file 1 [file cimb-47-00449-s001.zip › Figure S4A.png]

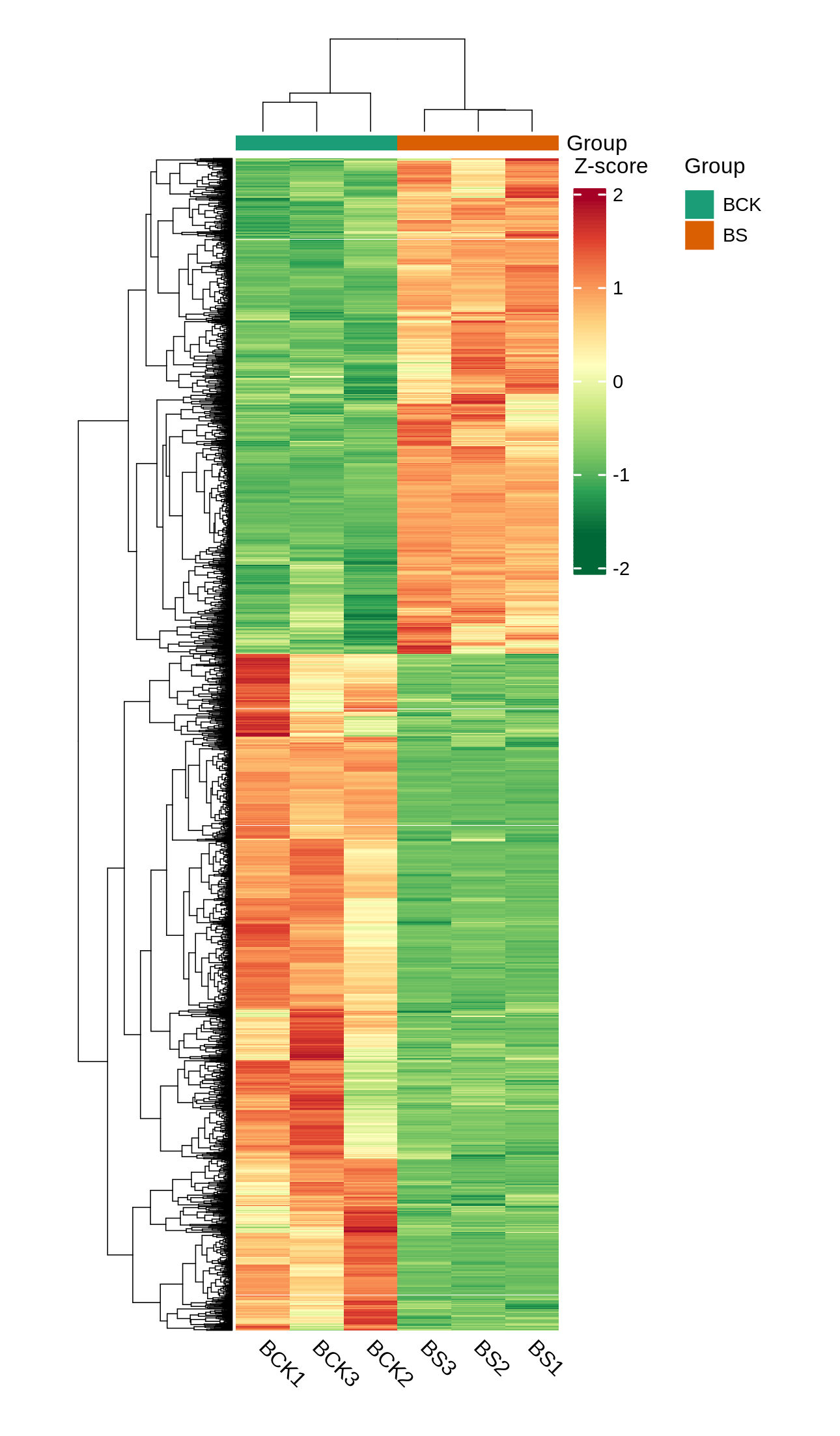

Supplement: Supplementary file 1 [file cimb-47-00449-s001.zip › Figure S4B.png]
